# Neuroprotective Effects of Soy Isoflavones on Scopolamine-Induced Amnesia in Mice

**DOI:** 10.3390/nu10070853

**Published:** 2018-06-30

**Authors:** Cong Lu, Yan Wang, Donghui Wang, Lijing Zhang, Jingwei Lv, Ning Jiang, Bei Fan, Xinmin Liu, Fengzhong Wang

**Affiliations:** 1 Institute of Food Science and Technology, Chinese Academy of Agricultural Sciences (CAAS), Beijing 100193, China; lucong198912@126.com (C.L.); wy198565@163.com (Y.W.); wangdonghui01@caas.cn (D.W.); zhanglijingg@126.com (L.Z.); fanbei517@163.com (B.F.); 2 Research Center for Pharmacology & Toxicology, Institute of Medicinal Plant Development (IMPLAD), Chinese Academy of Medical Sciences (CAMS) and Peking Union Medical College (PUMC), Beijing 100193, China; jwlv000@163.com (J.L.); jiangning0603@163.com (N.J.); xmliu@implad.ac.cn (X.L.)

**Keywords:** soy isoflavones, scopolamine, learning and memory, cholinergic system function, oxidative stress, ERK/CREB/BDNF pathway

## Abstract

In the recent years, interest in soybean as a neuroprotective nutrient in the management of Alzheimer’s disease (AD) has increased and soy isoflavones (SI), as kinds of soybean phytochemicals, are thought to be biologically active components that confer this beneficial effect against neurodegenerative diseases. However, the neuroprotective effect of SI is not well understood. Therefore, the present study (30 days) was conducted to investigate the neuroprotective effects of soy isoflavones (SI) on scopolamine (SCOP)-induced memory impairments in Institute of Cancer Research (ICR) mice (aged 4 weeks) and to elucidate its underlying mechanisms of action. SI (40 mg/kg) administration improved the cognitive performance of SCOP-treated mice in an object location recognition task and the Morris water maze test. SI (40 mg/kg) administration significantly enhanced cholinergic system function and suppressed oxidative stress levels in the hippocampus of SCOP-treated mice. Furthermore, SI (40 mg/kg) treatment markedly upregulated the phosphorylation levels of extracellular signal-regulated kinase (ERK), cAMP response element-binding protein (CREB) and brain-derived neurotrophic factor (BDNF) expression levels in the hippocampus. Taken together, these results demonstrated that soy isoflavones exerted a significant neuroprotective effect on cognitive dysfunctions induced by scopolamine, suggesting that soy isoflavones could be a good candidate for possible treatment of neurodegenerative diseases, such as Alzheimer’s disease (AD).

## 1. Introduction

Alzheimer’s disease (AD) is a central neurodegenerative disease that is prominently age-dependent; the proportion of AD patients is gradually increasing as society ages around the world [1]. According to a World Health Organization (WHO) report in 2015, AD affects approximately 48 million people all over the world [2]. Though the etiology of AD has yet to be definitively elucidated, the main pathophysiological features of AD have been proposed as follows: the accumulation of senile plaque and neurofibrillary tangles and the degeneration of cholinergic neurons [3,4]. Currently, drugs used for the treatment of AD mainly contain four acetylcholinesterase inhibitors (tacrine, rivastigmine, galantamine, and donepezil) and one *N*-methyl-d-aspartate (NMDA) receptor agonist (memantine) [5]. Unfortunately, these drugs have several adverse effects due to non-selectively action on a variety of organ tissues, both centrally and peripherally [6]. Therefore, it is necessary to explore complementary and alternative treatments for AD which have more effective strategy with fewer adverse effects.

Soybean (*Glycine max*, family Leguminosae) is a legume that is abundant in indigenous isoflavones. Intake of soybean has been linked to many aspects mainly in lowering the incidences of cardiovascular disease, risk of ischemic stroke, and cholesterol levels that in turn reduce the incidence of arthrosclerosis [7,8]. Additionally, in the recent years, interest in soybean as a neuroprotective nutrient in the management of AD has increased [9]. The soy isoflavones (SI), which can be considerd soybean phytochemicals, are thought to be the biologically active components that possess this beneficial effect against neurodegenerative diseases [10]. Clinical studies have shown that SI improved the cognitive function of postmenopausal women and had beneficial effects on cognition in older adults, especially older adults with AD [11,12]. Preclinical evidence suggests the neuroprotective effects of SI on cognitive dysfunctions in an amyloid β rat model of AD [13,14] and an ovariectomized rat model [15,16]. Moreover, it had been reported that total soy isoflavones could ameliorate scopolamine-induced amnesia in a rat model through the cholinergic neuronal pathway and prevention of neuroinflammation [9]. However, the neuroprotective effects of soy isoflavones have not yet been fully studied.

Therefore, the purpose of the present study was to examine the effects of soy isoflavones on scopolamine-induced memory impairment in mice by the object location recognition task and Morris water maze test. In addition, to investigate possible mechanisms of the SI-mediated improvement of learning and memory impairment, the changes in cholinergic system function, oxidative stress biomarkers, and the memory-related signal transduction molecules in the hippocampus were measured.

## 2. Materials and Methods

### 2.1. Chemicals and Reagents

Soy isoflavones (SI) (purify > 80% by HPLC) was purchased from Yuanye Biological Technology Co., Ltd. (Shanghai, China) and its chemical composition mainly included genistin 53.82%, daidzin 15.49% and glycitin 12.63%. Scopolamine (SCOP) was purchased from Sigma Chemical Co., Ltd. (St. Louis, MO, USA) and donepezil hydrochloride (Aricept) (DNP) was obtained from Eisai (Ibaraki, Japan). Carboxy methyl cellulose sodium (CMC-Na) was purchased from Shenggong Biological Engineering Co., Ltd. (Shanghai, China). Acetylcholinesterase (AChE), choline acetyltransferase (ChAT), malondialdehyde (MDA) and glutathione (GSH) commercial kits were obtained from Jiancheng Biological Technology Co., Ltd. (Nanjing, Jiangsu, China). 

### 2.2. Vehicle

SCOP and DNP were diluted in normal saline (0.9%, NaCl). SI was suspended separately in 0.5% (w/v) CMC-Na to obtain concentrations of 10, 20 and 40 mg/kg. 

### 2.3. Experimental Animals

Sixty adult male Institute of Cancer Research (ICR) mice (weighing 20–22 g, aged 4 weeks) were obtained from the Vital River Co., Ltd. (Qualified No.: SCXK 2016-0006, Beijing, China). All experimental procedures were carried out under the approval and supervision of the Academy of Experimental Animal Center of the Institute of Medicinal Plant Development (Haidian district, Beijing, China) (No: 2017114-03) and in accordance with the National Institute of Health Guide for the Care and Use of Laboratory Animals (Bethesda, MD, USA). And all efforts were made to minimize the suffering of the animals. All animals were housed at a temperature controlled (23 ± 2) °C condition with humidity of 50% ± 10% and a 12 h light/dark cycle (lights on at 8 a.m.). They were allowed to have free access to food and water for one week before the beginning of the experiment. 

### 2.4. Design of Animal Experiment

**Figure 1:**
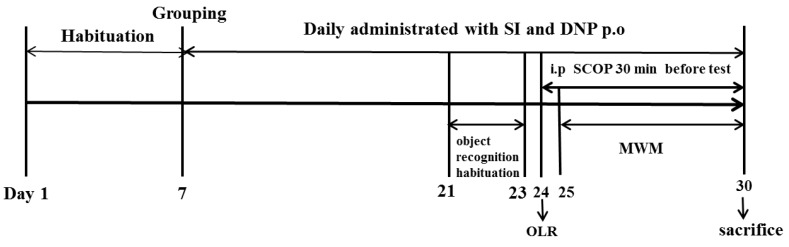
Timeline for the experimental procedure.

After the acclimatization period, the animals were randomly assigned to six groups (*n =* 10 in each group): Control group (Con); Scopolamine group (SCOP, 0.75 mg/kg); Soy isoflavones-L group (SI-10 mg/kg); Soy isoflavones-M group (SI-20 mg/kg); Soy isoflavones-H group (SI-40 mg/kg) and Donepezil group (DNP, 1.6 mg/kg). The number of mice per group used in the current study was determined according to the previous report [17]. The dose selections of SI, DNP and SCOP were based on the previous literature reports [9,18]. The animals in the SI and DNP groups received orally pretreatment with SI and DNP at corresponding concentrations, whereas the mice in the control and SCOP groups were treated orally with the equivalent volume of 0.5% CMC-Na for 13 days. After pretreatment, the mice in the SCOP, SI and DNP-treated groups were administrated intraperitoneally with SCOP (0.75 mg/kg) once daily for 7 consecutive days in order to induce memory impairment while the control mice received the same volume of normal saline by intraperitoneal administration. Thereafter, mice were subjected to memory assessment by the object location recognition (OLR) and Morris water maze (MWM). Following the behavioral tests, all animals were sacrificed and their hippocampus were isolated for further biochemical analysis. The whole study lasted for 30 days including the habituation period for 7 days, the pretreatment of SI and DNP for 13 days and the cognitive behavioral tests for 10 days (Figure 1). 

### 2.5. Object Location Recognition (OLR) Test

The object location recognition (OLR) experiment was performed to evaluate the short-term, spatial memory and the apparatus and procedures have been previously described [19]. Briefly, the task took place in a 40 cm × 50 cm × 50 cm rectangular box which was made of a black polyester plastic material and camera mounted on the top of the chamber to record animals’ exploratory behavior. Small, washable plastic material different in color but identical in shape and size (approximately 3 cm in diameter and 5 cm in height) were used as the sample objects (A1 and A2). The picture of A1 and A2 was shown in the Supplementary Material. The test was performed in three stages: habituation, familiarization, and test phases. Firstly, in order to habituate and reduce the animals’ fear of a new environment, mice were exposed to the apparatus with no objects and freely explore the surroundings for 10 min, which lasted for three consecutive days. Then on the fourth day, the mouse was placed into the chamber with two sample objects (A1 and A2) and was allowed to freely explore the objects for 5 min. After the familiarization phase, the mouse was returned to the holding cage, which remained inside the testing room. The exploration time for A1 and A2 object and the total exploration time (Te) were recorded in the familiarization phase. Finally, a test trial was conducted after a 30 min delay. During the test phase, mice were returned to the chamber in which one of the original objects had changed location (“novel”) and the other object remained in the original position (“familiar”). Objects and their placement into the arena were varied counterbalanced between groups to avoid positional biases. To control for possible odor cues, the objects and the floor of the arena were cleaned with 70% ethanol at the end of each trial to eliminate possible scent/trail markers. Exploratory behavior was considered only when the mice were sniffing or touching the object with the nose. In each trial, the duration of contact with each object was recorded using a stopwatch. Recognition memory was evaluated using a discrimination index (DI) calculated for each animal using the formula: DI = (TN − TF)/(TN + TF) [TN = time spent exploring the “novel” object; TF = time spent exploring the “familiar” object]. 

### 2.6. Morris Water Maze (MWM) Task

Morris water maze task (MWM) was then carried out to evaluate the long-term, spatial reference memory in mice. The mice were required to learn and locate a hidden platform just beneath the surface of a circular pool of water and also to remember its location in this task [20]. The protocol of MWM was conducted according to a published method [21]. The water maze was consisted of a circular, black pool measuring 1.0 m in diameter × 0.38 m in height, and filled with black ink at the temperature of (23 ± 2) °C to a depth of 25 cm. A video camera monitored the behavior of the mice in the pool. The procedure of MWM was comprised of the acquisition test for five consecutive days and the probe trial on the sixth day. During the acquisition test, mice were repeatedly placed into the tank and must learn to locate a hidden platform (6 cm in diameter) beneath in water. Each mouse was daily given 3 times into each of four quadrants in turn (except for target quadrant). Before each trial, mice were placed on the hidden platform for 10 s to be trained to remember the platform. Then they were gently released from one of four quadrants randomly and allowed to find the platform in 90 s. Learning and memory ability was measured by the escape latency. If mice did not locate the hidden platform within 90 s, the mice were gently guided to the platform, allowed to stay on the platform for 10 s, and the escape latency was recorded for 90 s. To assess spatial reference memory, the platform was removed and the probe trail was conducted after completion of the acquisition test. The animals were placed in the pool side opposite to the target (platform) quadrant and they were probed in a 90 s “retention” trial. Memory for the platform location was assessed by the virtual-platform crossing numbers. 

### 2.7. Brain Sample Preparation

After the behavioral tests, all mice were anesthetized by 10% chloral hydrate and decapitated rapidly. The hippocampus were dissected from the whole brain on ice respectively, separated from the midsagittal plane, and immediately snap-frozen and stored at −80 °C for further analysis. 

### 2.8. Biochemical Parameter Assay of the Hippocampus

For the biochemical detection, the hippocampus was weighed and homogenized in 9 vol (1:9, w/v) ice-cold 0.9% normal saline. After centrifugation at 3000 rpm for 10 min at 4 °C, the supernatant was collected and further diluted with appropriate buffer solution for the determination of the relevant biochemical parameters. AChE, ChAT, activities and contents of MDA and GSH in the hippocampus were measured using commercially available kits, respectively, according to the manufacturer’s instructions (Jiancheng, Nanjing, China). AChE activity was determined as described in the previous report [22]. Ellman’s reagent, 5′5-dithiobis (2-nitrobenzoate) is commonly known as DTNB. Briefly, Reaction mixture containing hippocampus homogenate (0.02 g/mL; 0.4 mL), 2.6 mL of phosphate buffer (0.1 M, pH 8.0), and 100 μL DTNB was mixed by bubbling air and placing it in a spectrophotometer. Once the reaction content was stable, absorbance was noted at 412 nm for the basal reading followed by addition of 5.2 μL of ATC to this cuvette. Any change in absorbance was recorded from zero time followed by 10 min at 25 °C. The ChAT activity was measured according to a published method [17]. The supernatant was incubated with reaction buffer (PBS pH 7.2; 6.2 mM acetyl coenzyme A; 1 M choline chloride, neostigmine sulfate 0.76 mM; 3 M NaCl; 1.1 mM EDTA). This mixture is added to 1 mM 4,4′-dithiodipyridine (4-PDS) and the absorbance read for 20 min at 324 nm in a microplate reader SpectraMax^®^ Microplate Reader (Molecular Devices^®^, Silicon Valley, CA, USA). MDA level was measured as described before [23]. For determination of MDA concentration (thiobarbituric acid reactive substances, TBARS), trichloroacetic acid and TBARS reagent were added to supernatant, then mixed and incubated at boiling water for 90 min. After cooling on ice, samples were centrifuged at 1000× *g* for 10 min and the absorbance of the supernatant was read at 535 nm. The results were obtained on tetraethoxypropane standard curve. GSH content was measured as previously reported [24]. The supernatant was centrifuged with 5% trichloroacetic acid. To 0.1 mL of homogenate, 2 mL of phosphate buffer (pH 8.4), 0.5 mL of DTNB and 0.4 mL of distilled water was added and the absorbance was read at 412 nm. 

### 2.9. Western Blotting Analysis

The Western blotting method was performed with slight modification according to our published procedure [25]. The hippocampus was homogenized on ice in CellLytic™ MT mammalian tissue lysis reagent (Sigma-Aldrich, St. Louis, MO, USA, C3228) containing protease inhibitor cocktail and phosphatase inhibitor cocktail (Sigma-Aldrich, St. Louis, MO, USA, P3840). The homogenate was centrifuged at 10,000× *g* for 15 min at 4 °C. Protein concentration in the supernatant was quantified by bicinchoninic acid (BCA) assay. Protein samples (30 μg/sample) were electrophoresed on a 10% sodium dodecyl sulfate polyacrylamide gel electrophoresis (SDS-PAGE) and transferred onto polyvinylidene difluoride (PVDF) membranes (Millipore, Burlington, MA, USA). The membrane was blocked with 5% nonfat dried milk at room temperature for 1 h followed by incubation with respective primary antibodies against phosphor ERK1/2, ERK 1/2, phosphor CREB, and CREB, BDNF (1:500), and β-actin (Cell Signaling Technology, Danvers, MA, USA) overnight at 4 °C. After thoroughly washed with PBST (phosphate buffered solution (PBS) with 0.1% Tween 20), the membrane was incubated with Horseradish peroxidase (HRP)-conjugated secondary antibodies at room temperature for another 1 h. The protein bands were visualized with ECL prime kit (GE Healthcare life Sciences, Shanghai, China).

### 2.10. Statistical Analysis

All data were analyzed using the SPSS 19.0 software package (Chicago, IL, USA) and expressed as means ± standard error mean (SEM). The data recorded from the acquisition trials of MWM among the groups over a period of five days were analyzed using repeated-measure two-way ANOVA. Paired *T*-test was conducted to compare the exploration time for the object A1 and the object A2 in the familiarization phase of OLR. The other data were analyzed by one-way ANOVA followed by multiple post hoc comparisons using the LSD test. The results of statistical analysis were performed with GraphPad Prism software 5.0 (GraphPad Software, La Jolla, CA, USA). For all statistical tests, *p* < 0.05 was regarded as significant.

## 3. Results

### 3.1. Effects of SI on Short-Term, Spatial Learning and Memory Deficits in the OLR Test

**Figure 2:**
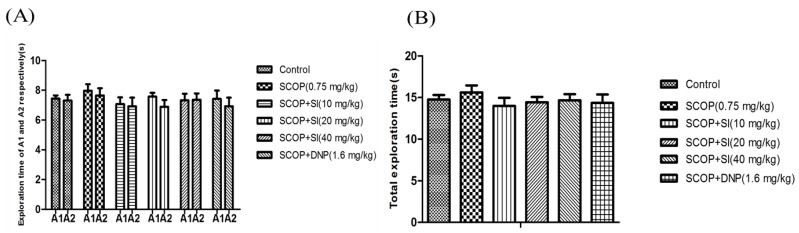
Effects of SI on the ability of exploration of SCOP-treated mice in the familiarization phase of OLR test. (**A**) Exploration time of A1 and A2 respectively of mice; (**B**) Total exploration time. Data are expressed as means ± SEM, *n =* 10 in each group.

**Figure 3:**
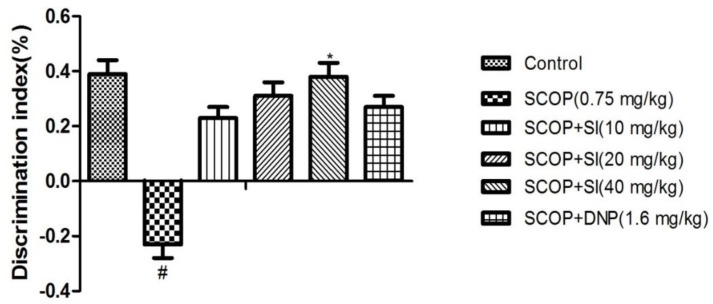
Effects of SI on short-term, spatial memory of SCOP-treated mice in the test phase of OLR test. Data are expressed as means ± SEM, *n =* 10 in each group. # *p* < 0.05 compared with the control group, * *p* < 0.05 compared with the SCOP group.

To evaluate the effects of SI on short-term, spatial memory in scopolamine-induced amnesia, the OLR test was conducted (Figure 2 and Figure 3). In the familiarization phase, the paired sample *T*-tests showed that there was no differences in the exploration time for the object A1 and the object A2 comparing between groups (Figure 2A), and analysis of the performance demonstrated that no clear differences were found between treatment conditions in the level of exploration (Figure 2B). The results ruled out the potential that there were any differences in the animals’ preference for location and the ability for exploration. 

In the test phase, when comparing groups, the effect of SI on the discrimination index (DI) was presented in Figure 3. The DI of the SCOP group was significantly lower than that of control group (*p* < 0.05). However, treatment with SI (40 mg/kg) significantly elevated the DI (*p* < 0.05) compared to the SCOP group, showing a short-term, spatial memory-improving effect. 

### 3.2. Effects of SI on Long-Term, Spatial Reference Memory in the MWM Task

**Figure 4:**
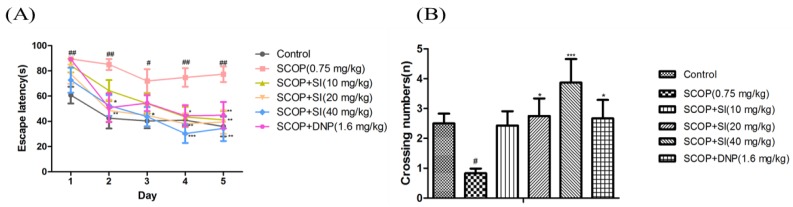
Effects of SI on the long-term, spatial reference memory of SCOP-treated mice in MWM task. (**A**) Escape latency in acquisition phase; (**B**) The crossing numbers in probe trial. Data are expressed as means ± SEM, *n =* 10 in each group. # *p* < 0.05, ## *p* < 0.01 compared with control group, * *p* < 0.05, *** *p* < 0.001 compared with the SCOP group.

Effects of SI on long-term, spatial cognitive abilities of SCOP-treated mice were determined by the MWM task (Figure 4). In the acquisition test, as shown in Figure 4A, the escape latency to locate the hidden platform gradually decreased during the acquisition test in all groups; however, the SCOP group exhibited significantly longer escape latency compared to the control group from day 1 to day 5 (*p* < 0.05, *p* < 0.01, respectively), illustrating the memory impairment successfully induced by SCOP treatment. SI (20 mg/kg) and SI (40 mg/kg) treatments decreased the escape latency from day 2 to day 5 compared to SCOP treatment with significance (*p* < 0.05, *p* < 0.01, *p* < 0.001, respectively). And SI (40 mg/kg) administration decreased the escape latency in the SCOP-treated mice more obviously compared to SI (20 mg/kg) treatment from day 3 to day 5. Moreover, SI (10 mg/kg) administration effectively decreased the escape latency on the fourth and fifth day (*p* < 0.05, *p* < 0.01, respectively). Treatment with DNP (1.6 mg/kg) also significantly improved SCOP-induced increments in escape latency on the second, fourth and fifth day (*p* < 0.01, *p* < 0.05, *p* < 0.01, respectively). 

In the probe test, the virtual-platform crossing number was used to evaluate retention of long-term, spatial memory. As shown in Figure 4B, the SCOP treatment significantly decreased the crossing numbers compared to the control group (*p* < 0.05). SI (20 mg/kg), SI (40 mg/kg) and DNP (1.6 mg/kg) treatment groups significantly increased the crossing numbers when compared with the SCOP group (*p* < 0.05, *p* < 0.001, *p* < 0.05, respectively). These findings demonstrated that SI administration significantly improved the long-term, spatial memory in SCOP-treated mice. 

### 3.3. Effects of SI on the Function of Cholinergic System in the Hippocampus of SCOP-Treated Mice

**Figure 5:**
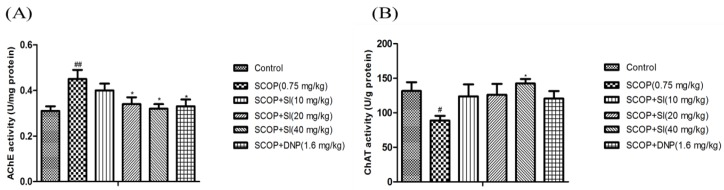
Effects of SI on AChE and ChAT activities in the hippocampus of SCOP-treated mice. (**A**) AChE activity; (**B**) ChAT activity. Data are expressed as means ± SEM, *n =* 10 in each group. # *p* < 0.05, ## *p* < 0.01 compared with the control group, * *p* < 0.05 compared with the SCOP group.

As shown in Figure 5A, the hippocampal AChE activity in the SCOP group was significantly higher than in the control group (*p* < 0.01). However, SI (20 mg/kg), SI (40 mg/kg) and DNP (1.6 mg/kg) treatments markedly decreased the AChE activity compared to the SCOP group (*p* < 0.05, *p* < 0.05, *p* < 0.05, respectively). The ChAT activity in the hippocampus of the SCOP group was significantly lower than that in the control group (*p* < 0.05), while SI (40 mg/kg) administration significantly increased the ChAT activity compared to the SCOP group (*p* < 0.05, Figure 5B). These results showed that SI treatment prevented SCOP-induced elevation of AChE activity and decrease of ChAT activity, which indicated that SI treatment could enhance the function of the cholinergic system.

### 3.4. Effects of SI on Oxidative Stress Biomarkers in the Hippocampus of SCOP-Treated Mice

**Figure 6:**
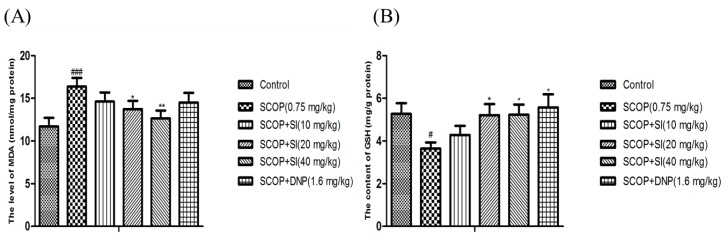
Effects of SI on MDA level and GSH content in the hippocampus of SCOP-treated mice. (**A**) MDA level; (**B**) GSH content. Data are expressed as means ± SEM, *n =* 10 in each group. # *p* < 0.05, *### p* < 0.001 compared with the control group, * *p* < 0.05, ** *p* < 0.01 compared with the SCOP group.

As shown in Figure 6A, the level of MDA was significantly increased in the hippocampus of SCOP-treated mice compared to the control group (*p* < 0.001). However, administration with SI (20 mg/kg and 40 mg/kg) significantly reduced the MDA increase induced by SCOP treatment (*p* < 0.05, *p* < 0.01, respectively, Figure 6A). Moreover, compared with the control group, the SCOP group had significantly lower GSH level (*p* < 0.05, Figure 6B), whereas significant enhancements on the GSH level were observed in SI (20 mg/kg), SI (40 mg/kg) and DNP (1.6 mg/kg) administration mice compared to the SCOP-treated mice (*p* < 0.05, *p* < 0.05, *p* < 0.05, respectively, Figure 6C). Overall, these results suggested that SI administration could inhibit the lipid peroxidation and promote the antioxidant level, indicating that SI may protect the hippocampus from oxidative stress damage induced by SCOP treatment.

### 3.5. Effects of SI on the Expression Levels of Memory-Related Molecules in the Hippocampus of SCOP-Treated Mice

**Figure 7:**
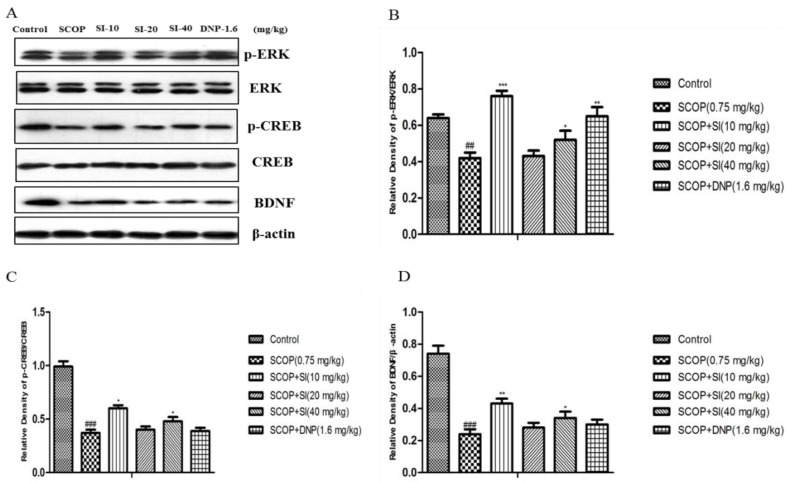
Effects of SI on p-ERK, ERK, p-CREB, CREB and BDNF expression levels in the hippocampus of SCOP-treated mice. (**A**) Western blot of p-ERK, ERK, p-CREB, CREB, BDNF and β-actin; (**B**) Gray intensity analysis of p-ERK/ERK; (**C**) Gray intensity analysis of p-CREB/CREB; (**D**) Gray intensity analysis of BDNF/β-actin. Data are expressed as means ± SEM, *n =* 3 in each group. ## *p* < 0.01, *### p* < 0.001 compared with the control group, * *p* < 0.05, ** *p* < 0.01 *** *p* < 0.001 compared with the SCOP group.

To assess the effects of SI on the levels of memory-related proteins in the hippocampus, Western blotting analysis was performed. As shown in Figure 7, the SCOP group had significantly lower protein levels of phosphorylation of ERK, phosphorylation of CREB and BDNF in the hippocampus compared to control group (*p* < 0.001, *p* < 0.001, *p* < 0.001, respectively). SI (10 mg/kg) and SI (40 mg/kg) administration significantly increased phospho-ERK, phospho-CREB and BDNF levels compared with the SCOP group (*p* < 0.001, *p* < 0.05, *p* < 0.01, respectively). In addition, DNP (1.6 mg/kg) dramatically reversed the decrease in protein levels of phospho-ERK induced by SCOP treatment (*p* < 0.01, *p* < 0.05, respectively).

## 4. Discussion

As the incidence of AD continues to rise very rapidly [26], research on effective treatments for AD would be valuable. Accumulating evidence suggests that naturally occurring phytochemicals may potentially delay neurodegeneration, and improve cognitive function [27,28]. The effects of these compounds have attracted research in this field in order to assess their efficacy as nootropic agents [29]. Soy isoflavones are among the important phytochemicals in soy and soy products that are recognized and most studied for their well-established positive effects against chronic diseases [30]. In the present study, we evaluated the memory improvement effects of soy isoflavones on scopolamine-induced cognitive dysfunction in mice using the object location recognition test and the Morris water maze task. Additionally, to elucidate the underlying mechanisms of SI, we examined cholinergic system function, oxidative stress biomarkers and memory-related signaling pathways in the hippocampus of mice.

To assess the effects of SI on cognitive function in the scopolamine mouse model, two types of cognitive behavioral tests were conducted. Each test represented a different role in cognitive functions: Object location recognition (OLR) test was used to evaluate the short-term, spatial memory ability of mice [31]. It takes advantage of the rodent spontaneity to explore “novel” objects in a relatively short time [32]. Our present study observed that the mice treated with SCOP (0.75 mg/kg) could not distinguish the “novel” object (changed location) relative to “old” object (unchanged location), and a significant reduction of the discrimination index (DI) was found compared to the control group, which was consistent with our previous report [17]. Moreover, for the first time, we found that SI (40 mg/kg) treatment significantly improved the impaired object location recognition memory in SCOP-treated mice, indicating by the increased DI and it suggested SI could enhance impaired short-term, spatial memory in SCOP-treated mice. The Morris water maze (MWM) task is a hippocampus-dependent memory task frequently used in rodents for evaluating long-term spatial and reference memory [33,34]. Our current finding that SCOP (0.75 mg/kg) administration to mice significantly prolonged the escape latency and reduced the crossing numbers over a platform location compared with the control group was consistent with previous studies, demonstrating cognitive dysfunction in the long-term, spatial learning and memory ability [25,35]. However, SI administration improved learning and memory performance as indicated by a significant decrease in the escape latency as well as increasing the crossing numbers in the MWM test compared with the SCOP-treated mice, exerting the positive effect on the impaired long-term spatial and reference memory induced by SCOP treatment. The results were similar to the previously reported cognitive enhancement effects of total soy isoflavones at the dose of 40 mg/kg in the scopolamine-induced amnesia model [9]. Taken together, these behavioral tests demonstrated that SI treatment effectively improved the impaired learning and memory in SCOP-treated mice.

The central cholinergic system plays a vital role in memory processes [36]. A major factor in age-related senile central nervous system dysfunction and the early stages of AD may be a disruption in the cholinergic neurotransmission system [37]. Scopolamine is a non-selective muscarinic acetylcholine receptor antagonist and it has been reported to be associated with learning and memory impairments by inhibition of the cholinergic system in the central nervous system [38]. Thus, in order to explore the possible mechanisms of memory-enhancing effects of SI in SCOP-treated mice, the change of cholinergic system was evaluated. Our results together with those of other reports [39,40] showed that increased AChE activity and decreased ChAT activity in the hippocampus were observed in the SCOP-treated mice. Treatment with SI reversed SCOP-induced increase of AChE activity and decrease of ChAT activity in mouse hippocampus, which was consistent with the previous study [9]. Therefore, these results suggested that the ameliorative effects of SI on SCOP-induced memory impairment could be explained by their modification of the cholinergic system function.

A large body of evidence suggested that oxidative stress is another causative factor involved in age-related neurodegenerative disorders, which include AD [6,41]. The amnesia induced by SCOP had been reported to be associated with the increase of oxidative stress in the brain tissue, especially the hippocampus [42]. In agreement, our present study showed that SCOP treatment resulted in remarkable oxidative stress damage in the hippocampus as evidenced by the decreased antioxidant defense systems such as GSH levels and a significant elevation of MDA levels which presented as a valid index of lipid peroxidation. However, SI administration robustly restored elevated MDA levels and decrease of GSH content in SCOP-treated mice, showing a beneficial role in the reduction of oxidative stress. It was similar to the previous founding that effect of SI on learning and memory ability of chronically Al=exposed animals might be related to their anti-oxidative function [10]. Besides, it has been demonstrated that SI has antioxidant properties via detoxifying free radical species and upregulating antioxidant genes [43]. Hence, it suggested that the reduction of oxidative stress could be related to the cognitive effect of SI in SCOP-treated mice.

Extracellular signal-regulated kinase (ERK1/2) is a kinase involved in the consolidation of spatial memory and its activation is necessary for the establishment of long-term potentiation (LTP), which is associated with neuronal plasticity and development of memory [44]. ERK is involved in the LTP-dependent transcriptional regulation through activation of transcriptional factor cAMP response element binding (CREB) in hippocampal regions. Moreover, CREB phosphorylation is essentially required for memory formation and storage in the hippocampus. Brain-derived neurotrophic factor (BDNF) is a key downstream target of CREB signaling and is important in the development and survival of neurons as well as in regulation of synaptic transmission in the hippocampus [45]. Thus, it has been suggested that the activation of the ERK/CREB/BDNF signaling pathway may be involved in the enhancement of memory performance [46]. Accumulating data has suggested that ERK phosphorylation, CREB phosphorylation and BDNF expression in the hippocampus of murine brains are inhibited by treatment with SCOP [47,48]. Our current results were consistent with previous studies and to the best of our knowledge it was the first time that SI treatment was found to upregulate p-ERK, p-CREB and BDNF expression levels in SCOP-treated mice. It is suggested that the protective effect of SI on SCOP-induced memory deficits may be partly due to the modulation of the ERK/CREB/BDNF signaling pathway.

Limitations still exist in the current study. It was reported that ingested soy isoflavones reaches concentrations sufficient to elicit physiological actions in the periphery and central nervous system [49], however, more insight into the pharmacokinetic properties of SI especially for that the blood–brain barrier permeability of SI could be a useful tool in its development as a suitable treatment and should be considered in in vivo studies. In addition, the exact and intensive mechanisms of the neuroprotective effects of SI need to be elucidated in further studies.

## 5. Conclusions

In conclusion, the present study revealed that soy isoflavones have neuroprotective effects on scopolamine-induced memory impairments in mice and these effects may be partially due to the enhancement of cholinergic system function, suppression of oxidative stress and activation of the ERK/CREB/BDNF signaling pathway. Therefore, it suggested that soy isoflavones may be potential candidates for the treatment of neurodegenerative diseases, such as Alzheimer’s disease (AD). Thereafter, more different types of behavioral tasks, more cognitive impairment animal models and the specific mechanisms of SI need to be designed in the future for elucidating the neuroprotective effects of SI.

## Supplementary Materials

The following are available online at http://www.mdpi.com/2072-6643/10/7/853/s1.
