# Supplementary figures and images for: Neuroprotective Effects of Soy Isoflavones on Scopolamine-Induced Amnesia in Mice
Source: Nutrients. 2018 Jun 30;10(7):853. doi: 10.3390/nu10070853 (PMC6073222; doi:10.3390/nu10070853)

A1

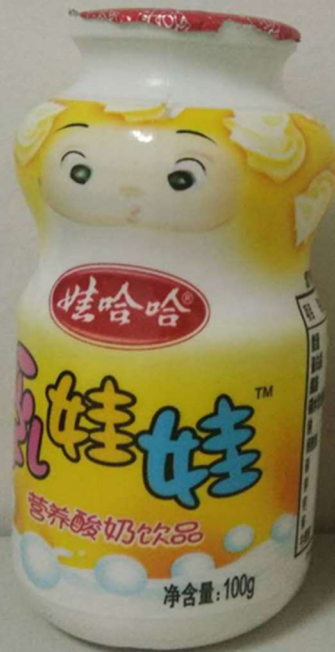

A2

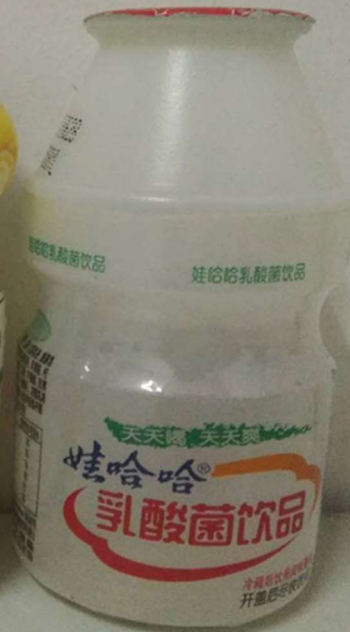

Supplement: Supplementary file 1 [file nutrients-10-00853-s001.pdf]
